# Daycare Center Attendance Buffers the Effects of Maternal Authoritarian Parenting Style on Physical Aggression in Children

**DOI:** 10.3389/fpsyg.2017.00391

**Published:** 2017-03-21

**Authors:** José M. Muñoz, Paloma Braza, Rosario Carreras, Francisco Braza, Aitziber Azurmendi, Eider Pascual-Sagastizábal, Jaione Cardas, José R. Sánchez-Martín

**Affiliations:** ^1^Psychology Department, Faculty of Sciences of Education, University of Cadiz, Puerto RealSpain; ^2^Doñana Biological Station, Spanish Council for Scientific ResearchSevilla, Spain; ^3^Department of Basic Psychological Processes and their Development, Faculty of Psychology, University of the Basque CountrySan Sebastian, Spain; ^4^Department of Developmental and Educational Psychology, University of the Basque CountryLeioa, Spain; ^5^Department of Psychology and Pedagogy, The Public University of NavarrePamplona, Spain

**Keywords:** daycare center attendance, mother authoritarian style, physical aggression, sex differences, kindergarten

## Abstract

A maternal authoritarian style has been related to the development of physical aggression during childhood and later future social problems; however, not too many studies have detected other than individual or family factors that may buffer this maternal effect. This work examines whether daycare center attendance may moderate the relationships between a mother authoritarian style and physical aggression. The study sample was 72 (40 girls) kindergarten children from Spain. Parents were asked to complete two questionnaires focused on individual family characteristics and parenting styles. At age 5, children physical aggression was assessed by direct observation at playtime; aggression scores at 6 was obtained by a peer-rated questionnaire. A least squared multiple regression was performed after controlling for children’s level of physical aggression at 5, child sex and siblings. A positive contribution of maternal authoritarian style on physical aggression was detected. Daycare center attendance appears to attenuate the effect of the mother’s authoritarian style on physical aggression, only in boys.

## Introduction

Empirical research confirms that children frequently engaged in physically aggressive behavior adopt a style of responding to interpersonal conflicts that leads them to develop more serious forms of maladjustment such as poor relationships, violence, and delinquency (Card et al., 2008; Rubin et al., 2009). Besides, research evidence indicates that children’s early aggressive responses, and their relations with parents and out-of-home care experiences may all contribute to later behavioral problems (Brame et al., 2001; NICHD Early Child Care Research Network, 2003; Li et al., 2011).

First, despite evidence suggesting that aggressive behaviors may be adaptive (Archer and Côté, 2005), atypically high levels of aggression in early childhood, especially physical aggression, are generally associated with a wide range of social problems (Nagin and Tremblay, 2001; Di Giunta et al., 2010). Most preschoolers use physical aggression, but only a small proportion of individuals are persistently physically aggressive (Brame et al., 2001; Broidy et al., 2003) what may represent a risk for future difficulties. Although boys tend to be more physically aggressive than girls (Barth et al., 2004; Rose and Rudolph, 2006), longitudinal studies show that persistence of physical aggressive behavior from an early age is not different among both boys and girls (Broidy et al., 2003; Borge et al., 2004; Côté et al., 2007).

Secondly, many studies research how parenting influences children’s aggressive behavior. Physical punishment and harsh discipline were proven significantly related to child’s aggressive behavior (see Deater-Deckard and Dodge, 1997; Deater-Deckard et al., 1998; Gilliom and Shaw, 2004; Alink et al., 2009; Tanaka et al., 2010). Most research on associations between negative parenting style and child’s development of behavioral problems is focused on mothers, because they have traditionally been assumed to spend more time, on average, than fathers in direct interactions with their young children, and most researchers agree that mothers usually play a central role in child development (see Bornstein, 2006). In fact, some authors (e.g., Miner and Clarke-Stewart, 2008; Wang et al., 2013) observe that precisely maternal harsh discipline is significantly related to the frequency of children externalizing behavior, characterized by aggression, defiance, and acting-out attitudes. A recent study by Braza et al. (2015) focused on the effects of negative maternal and paternal parenting styles on child’s aggressive and behavioral problems shows that only maternal authoritarian style (characterized by a high level of perceived hostility, punishment, restrictiveness, and intrusiveness) contributes directly to the development of these risk aggressive behaviors, regardless of fathers’ parenting style. Besides, empirical evidence shows a different effect in boys and girls of parenting style on physical aggression (Casas et al., 2006; Braza et al., 2015).

Regarding the relation between out-of-home experiences and aggression in children, most studies research associations by means of broad measures of behavioral problems and different types of aggressive responses (Clarke-Stewart et al., 1995; NICHD Early Child Care Research Network, 2002). However, as Côté et al. (2008) point out, few studies have examined specifically these associations with physical aggression; the results of these scarce studies focused on physical aggression are contradictory. Haskins (1985) found that high-risk African American children whose non-maternal care began early in their lives are more physically aggressive, while NICHD Early Child Care Research Network (2004) found that childcare is not systematically associated with physical aggression during childhood. On the other hand, Crockenberg (2003) points out that child sex—together with their type, quality, and amount of care—is likely to influence differences in child development, and should not be overlooked in studies on childcare effects. Nevertheless, these sex differences are not usually taken into account in studies focused on the association of out-of-home experiences and aggression in children.

Due children’s early aggressive behavior, negative maternal style and day care center attendance are relevant factors to future aggressive trajectories, is necessary that research continue examining simultaneously the influence of all these variables on physical aggression in children. In this sense, Côté et al. (2007) have already suggested that non-maternal care can reduce the risk of a high physical aggression trajectory from infancy to school. This research studies maternal authoritarian style as predictor of physical aggression in boys and girls at the age of 6.

Although there are clear links between the positive involvement a child enjoys with a sibling and peer competence, it is also the case that sibling relationships can contribute to the development of peer aggression (Volling and Blandon, 2005). Several studies have found relations between the aggression between siblings and the children’s use of aggression with peers (e.g., Stormshak et al., 1996; MacKinnon-Lewis et al., 1997). Even as toddlers, physical aggression by an older sibling directed toward an old younger sibling predicted the use of physical aggression by the younger sibling 6 months later (Dunn and Munn, 1986). So, since evidence shows that early aggression and having a sibling have been associated with a higher risk of physical aggression in childhood (Tremblay et al., 2004), both physical aggression at age 5 and siblings are controlled in this study.

Daycare attendance is expected to be a buffering protective factor in the associations between maternal authoritarian style and physical aggression. Due maternal authoritarian style was shown a risk factor for the development of aggressive behavior (Braza et al., 2015), we expect that Spanish daycare center, characterized by a positive classroom atmosphere (Sandstrom, 2012), may buffer this negative effect.

Specifically, the present study tested the following hypotheses:

(1) Maternal authoritarian style increases physical aggression during childhood;
(2) Daycare center attendance buffers the effect of maternal authoritarian style on physical aggression; and
(3) This buffering effect differs from boys to girls.

## Materials and Methods

### Participants

The sample consists of 72 children (32 boys and 40 girls) from three Spanish public schools. Their mean age at the beginning of the study was 63.02 months (SD = 3.3). In Spain, a free preschool education period is provided to all children willing to enroll. Subsidized educational services are provided to nearly 100% of 3- to-5-year-old children, above any other European country (see Sandstrom, 2012, for further information). Children were assessed at kindergarten and first grade. The study was explained to the participating schools’ directors and teachers, and participating children’s parents, and their written informed consent was requested. A total of 127 two-parent families of medium socioeconomic status gave their informed consent; during the period of study, the participants who did not attend a day of testing, provided incomplete data or moved away from the area were excluded from analyses. The final ratio of participants in each classroom was over 60% relative to the initial sample. Although a non-invasive test was used, the project was pre-approved by the Ethics Committee of the institution the authors belong to.

### Procedure

Parents were asked to complete two questionnaires upon daycare enrolment—one focused on the individual characteristics of their family members and socioeconomic circumstances, and another one aimed at assessing their parenting styles (Parenting Styles and Dimensions Questionnaire, PSDQ; see Robinson et al., 1995, 2001). Physical aggression at the age of 5 was assessed by direct observation at playtime in daycare time, when children were observed interacting freely with their peers. Aggression scores at the ages of 6 was obtained at the beginning of the first grade by a peer-rated questionnaire.

Peer ratings were collected in an individual interview using a Likert scale that asked participants to rate the frequency each of their same sex classmates displays physical aggressive behaviors (hitting, kicking, tripping, etc.).

### Measures

#### Predictor Variable: Maternal Authoritarian Style

Each mother received her own PSDQ questionnaire packet directly from their child’s school. The instrument contains 62 statements regarding different parent reactions to child behavior. Items use a 5-point Likert scale ranging from never (1) to always (5). This instrument is aimed at measuring parenting styles along Baumrind’s (1989) continuum of typologies: authoritative, authoritarian, and permissive. The measure yields a separate, continuous score for each parenting dimension, with higher numbers indicating increased use of parenting practices associated with a particular style. Only the overall scale for authoritarianism was used in the analyses in this study; this scale include items like “uses physical punishment as a way of disciplining our child” (corporal punishment factor), “yells or shouts when child misbehaves” (verbal hostility factor), “punishes by taking privileges away from child with little if any explanations” (non-reasoning, punitive strategies factor) or “tells child what to do” (directiveness factor). PSDQ has been praised in a review of instruments for parenting practice assessment (Locke and Prinz, 2002) as one of the few available instruments with psychometrically defensible scales regarding parental nurturance and discipline.

#### Moderator Variables: Daycare Center Attendance and Child Sex

Also in the same family characteristics questionnaire, daycare center attendance was run as a categorical variable reflecting attendance (1; *N* = 56; 24 boys) or absence (0). Daycare center attendance refers to children attended in public daycare centers for 5 h a day since they are 3–4 years old. Data for boys and girls were analyzed separately.

#### Behavioral Outcome

Physical aggression at the age 6 was assessed and measured using an interview version of the Direct and Indirect Aggression Scale (DIAS) by Björkqvist et al. (1992), a peer rating measurement instrument for aggressive behavior. DIAS is a test containing 24 items asking children to rate each of their same-sex classmates on a 5-point Likert scale (0–4) regarding aggression-related behaviors. The final scores in the physical aggression scale (including items like “hits the other one?” or “takes things from the other one?”) were obtained by summing up the scores for each item, and then dividing this total sum by the number of items that make up each scale. This subscale proves reliable for this sample (Cronbach’s α: 0.96).

#### Confounding Variable

Physical aggression at age 5 was measured by direct observation at open-air playgrounds where children could interact freely at playtime in the break, with no adult presence except for teachers’ surveillance from the playground entrance. Children were filmed at least twice a week with a video camera during the central 15 min of their daily 30-min playtime from November to June. Behaviors such as hitting, kicking, and pushing (Braza et al., 1994) were recorded using focal sampling and continuous recording methods (Martin and Bateson, 1986), and each child’s behavior was sequentially analyzed. Each participant was filmed for 2 min on a rota basis, no participant being filmed again until all the other participants on the list had already been filmed. This procedure resulted in a total number of 15 min of film for each child. Physical aggression analysis and quantification were completed using Observer 4.1 behavior analysis software. Two observers simultaneously coded the behaviors of 10 children three times during the study period. Agreement between both coders was assessed and any discrepancies were discussed. Agreement is never below 85%, and average agreement was 90.67%. Kappa was never below 0.80.

#### Confounding Variable

Siblings were included as a categorical variable because it can affect physical aggression levels in early childhood (Tremblay et al., 2004). Children with no siblings (0) were distinguished from those who had them (1).

### Statistical Analysis

Sex differences were analyzed by means a one-way ANOVA for physical aggression and by means a chi-square test for siblings and daycare center variables. The relationships between continuous variables were examined using a Pearson correlation coefficient. A one-way ANOVA was conducted to test differences in the level of maternal authoritarian style between the groups with and without daycare center attendance.

The moderating effect of daycare center attendance has been studied with multivariate methods in which confounding variables were controlled in regression analyses. So, least-squares multiple regression was performed to analyze the influence of child sex, maternal authoritarian style, and daycare on the development of physical aggressive behavior at the age of 6, after controlling for siblings and previous levels of physical aggression at the age of 5. In order to test whether the relationship between maternal authoritarian style and child physical aggression differs between groups of children with and without daycare center attendance, the interaction between maternal authoritarian style and daycare center attendance was included in the model. In this case, physical aggression was our criterion variable, level of authoritarian style is our predictor variable (both being quantitative variables), and daycare attendance is our moderator variable. Prior to forming the product terms, the quantitative predictor was standardized. Finally, the interactive effects of maternal authoritarian style and daycare attendance were analyzed separately for boys and girls.

## Results

### Preliminary Analyses

One-way ANOVAs revealed significant sex differences for physical aggression at the age of 6 [*F*(1,81) = 23.68, *p* < 0.0001, ηp^2^ = 0.231], with boys scoring higher than girls (*M* = 1.16, SD = 0.86 and *M* = 0.49, SD = 0.56). Sex differences regarding siblings and daycare center experience are not statistically significant (χ^2^ = 0.28; *p* = 0.5964; and χ^2^ = 0.30, *p* = 0.5861, respectively).

Pearson’s correlations revealed that maternal authoritarian style was associated with physical aggression at the age of 6 (*r* = 0.27, *p* < 0.05) for girls. ANOVA shows no significant differences (*p* = 0.066, ηp^2^ = 0.047) in the level of maternal authoritarian style between the groups with and without daycare center attendance. So, both groups (with and without daycare center attendance) are characterized by the same degree of risk (level of maternal authoritarian style).

### Predictors of Physical Aggression and the Moderating Effects of Daycare Center Attendance

Multiple regression analysis—controlling for physical aggression at the age of 5 and siblings—was performed on peer’s scores on physical aggression at the age of 6, main effects being child sex, daycare center attendance, and maternal authoritarian style—as well as interaction effects between these variables. As shown in **Table 1**, child sex and maternal authoritarian style contributed significantly to variance in peer-rated physical aggression at the age of 6. Moreover, interactions between maternal authoritarian style and daycare center attendance, as well as maternal authoritarian style and daycare center attendance and child sex, are statistically significant for physical aggression at the age of 6. As expected, this last interaction evidences a different sex-related moderating effect of the daycare center attendance on the relationship between maternal authoritarian style and physical aggression.

**Table 1:** Regression analysis for physical aggressive behavior at the age of 6.

	Physical aggression at the age of 6	
	*B*	Standard error
Child sex	0.49ˆ***	0.12
Physical aggression at the age of 5	-0.01	0.10
Siblings	-0.07	0.10
Maternal authoritarian style	0.43ˆ**	0.13
Daycare center attendance	-0.19	0.12
Mother authoritarian style × child sex	0.14	0.13
Daycare center attendance × child sex	0.12	0.12
Mother authoritarian style × daycare center attendance	0.34ˆ*	0.14
Mother authoritarian style × daycare center attendance × child sex	0.43ˆ**	0.13

*R^*2*^ = 0.49, *F*(9,72) = 6.70, *p* < 0.0001. ^∗^*p* < 0.05; ^∗∗^*p* < 0.01; ^∗∗∗^*p* < 0.001.*

**FIGURE 1:**
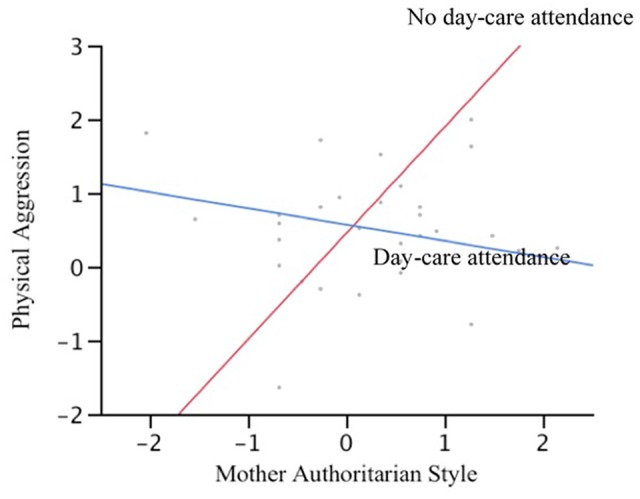
**Daycare center attendance as a moderator of the relationship between mother authoritarian style and boys’ physical aggression at the age of 6**.

The interaction of maternal authoritarian style and daycare center attendance was determined separately for girls and boys, by two multiple regression analyses (see **Table 2**). Only for boys, the interaction between maternal authoritarian style and daycare center attendance is shown to have a significant contribution to physical aggression. **Figure 1** shows that the influence of maternal authoritarian style on physical aggression is statistically significant for homeschooled boys but not for their counterparts attending daycare centers (β = 0.75, *p* = 0.0308, and β = -0.40, *p* = 0.0550, respectively).

**Table 2:** Hierarchical regression analysis for physical aggressive behavior at the age of 6.

	Physical aggression at the age of 6	
	*B*	Standard error
Girls		
Physical aggression at the age of 5	0.05	0.16
Siblings	-0.22	0.15
Maternal authoritarian style	0.29	0.21
Daycare center attendance	-0.30	0.22
Mother authoritarian style × daycare center attendance	-0.09	0.21
Boys		
Physical aggression at the age of 5	-0.06	0.12
Siblings	0.10	0.15
Maternal authoritarian style	0.64^∗∗^	0.18
Daycare center attendance	0.18	0.14
Mother authoritarian style × daycare center attendance	0.89^∗∗∗^	0.19

*For girls, *R*^*2*^ = 0.23, *F*(5,40) = 2.03, *p* = 0.0993; for boys *R*^*2*^ = 0.47, *F*(5,32) = 4.70, *p* = 0.0034. ^∗∗^*p* < 0.01; ^∗∗∗^*p* < 0.001.*

## Discussion

This study is aimed at examining the possible moderating role of daycare center attendance in 3- to 4-year-olds in the relationship between maternal authoritarian style and physical aggression at the age of 6. The main conclusions of our study are: (a) a maternal authoritarian style increases physical aggression; (b) daycare center attendance seems to have, only for boys, a buffering protective effect against the risk of a maternal authoritarian style.

Early physical aggression in children is associated with later antisocial behavior, however, according to several longitudinal studies most children follow a low-decreasing or no aggression profile (around 70%), while only around 4–10% of the sample follow a chronic physical aggression trajectory (Broidy et al., 2003; Chen et al., 2011), proving that children aggression is subjected to a heterogeneous developmental pattern over time. These differences in aggression trajectories may be explained by differences in developmental contexts (mainly family and school). Many studies indicate that negative parenting style involving hard punishment is related to later aggressive behaviors during childhood (Stormshak et al., 2000; Erath et al., 2006; Taylor et al., 2010).

Thus, given that maternal authoritarian style promotes the development of aggressive behaviors, it might be interesting to identify protective factors that buffer the effects of maternal authoritarian style on the development of physical aggression during childhood to develop intervention strategies.

Despite our limited sample, we decided to explore these buffering effects. The results of this study suggest that daycare center attendance from the ages of 3 to 4 may attenuate the effect of maternal authoritarian style on physical aggression development among boys. This buffering protective effect on physical aggression may be due to both the benefit of positive interactions with peers and caregivers others than their parents, and the reduced time children spend with their authoritarian mother. Play and positive peer interaction in preschool years predict social competence with peers in middle childhood (Hay et al., 2009), and promote child’s cognitive and emotional growth (see Singer et al., 2006). Interactions with caregivers in daycare center provides positive learning opportunities that are either not so readily available at home or higher quality than those received at home (Borge et al., 2004; Côté et al., 2007). Belsky et al. (2007) find these interactions have positive influences on child’s vocabulary, empathy, emotional regulation, and other socio-cognitive and emotional abilities that, in turn, could indirectly help to reduce their physical aggression levels. Moreover, an experimental intervention study shows that a reduction in the time children spend under harsh parental control predicts lower physical aggression rates in a sample of preschoolers in risk (Brotman et al., 2009). Mechanisms through which daycare center attendance buffers parenting’s negative effects on later aggression in boys should be studied in depth—e.g., through the provision of positive social interactions with same-age peers or caregivers’ promotion of early cognitive and language skills (Phillips et al., 2006).

On the other hand, no buffering effect of daycare attendance is detected in the relationship between maternal authoritarian style and physical aggression in girls. So, it seems that daycare center attendance does not attenuate girls’ physical aggression associated with a maternal authoritarian style. In despite the small size of the study sample, some explicative hypotheses are considered in relation with social learning theory (Bandura, 1973). This theory suggests that parental modeling of aggressive behaviors may underlie the relationships between child’s exposure to harsh discipline and aggressive behavior. This effect may be general or, alternatively, maternal or paternal harsh discipline may have partial or full sex-specific effects (i.e., maternal harsh discipline may have stronger effects on daughters than on sons). Some researchers point out that girls who have observed their mothers’ aggressive behavior toward their partners are significantly more aggressive toward their friends; and similarly, boys who witnessed their fathers’ aggression are also significantly more aggressive toward their friends (Moretti et al., 2006). In this line, other researchers suggest that while girls behave on the basis of observation of their mother’s role, boys behave on the basis of observation of another role such as their father’s or that of another influential family member (MacBrayer et al., 2003).

A more comprehensive explanation should take into account other individual or contextual (school and family) factors that might be candidates for having protective effects in relation to the negative influence from a maternal authoritarian style. Besides, a potential direct protective effect of daycare center attendance should be considered. Although our results do not have detected this direct protective effect, this may be due to the low amount of physical aggressive behavior in girls, compared with boys; probably a large sample is needed to explore this direct effect in girls. Further research and analysis of these factors shall elucidate the direct and buffering protective factor for a trajectory of aggressive behavior.

Although the majority of child care settings provide children with a warm, supportive environment that protects children’s health and safety, only a small percentage of children in child care receive caregiving which promotes and stimulates development (Doherty et al., 2000). Further research is needed to elucidate the quality of the day care organization, in order to define which day care attendance features are more protective; for instance, there is some evidence that child care centers that are inclusive (that welcome and accommodate children with special needs) tend to be of higher quality than non-inclusive programs (Buysse et al., 1999).

We are aware that this study bears some limitations; the sample size is not extensive enough and shows an uneven distribution of the moderator variable (daycare attendance); the study is correlational in nature and so caution is needed upon concluding causal relationships. Thus, the results should be interpreted in the light of these limitations.

Some of the strengths of the present study are the adoption of a sequential study design, the control for child’s personal characteristics (sex, siblings, and early level of physical aggression), and the availability of multiple informants’ reports (parents’, peers’, and direct observation) to study the observed variables.

To sum up, this study advances the existing conceptual and empirical knowledge on the effect of parenting practices on child’s future social-emotional adjustment. This study adds to the available literature on the importance of daycare center attendance in 3- to 4-year-olds to reduce the aggression generated by the effect of maternal authoritarian style. We think that daycare attendance prior to school is a more affordable and less costly strategy than other interventions within the family context.

## Author Contributions

All authors (JM, PB, RC, FB, AA, EP-S, JC, and JS-M) have participated in the various phases of work, from data collection to final wording and revision of the manuscript.

## Conflict of Interest Statement

The authors declare that the research was conducted in the absence of any commercial or financial relationships that could be construed as a potential conflict of interest.
